# Sequential Astrocytic 5-HT_2B_ Receptor Stimulation, [Ca^2+^]_i_ Regulation, Glycogenolysis, Glutamate Synthesis, and K^+^ Homeostasis are Similar but Not Identical in Learning and Mood Regulation

**DOI:** 10.3389/fnint.2015.00067

**Published:** 2016-01-08

**Authors:** Ye Chen, Ting Du, Liang Peng, Marie E. Gibbs, Leif Hertz

**Affiliations:** ^1^Henry M. Jackson FoundationBethesda, MD, USA; ^2^Laboratory of Metabolic Brain Diseases, Institute of Metabolic Disease Research and Drug Development, China Medical UniversityShenyang, China; ^3^Drug Discovery Biology, Monash Institute of Pharmaceutical Sciences, Monash UniversityParkville, VIC, Australia

**Keywords:** astrocytes, calcium, clozapine, fluoxetine, memory, psychiatric disorder

## Interrelation between anxiety and memory

A close interrelation between anxiety and memory was first suggested by Kalueff and Nutt (1996), reviewing effects of γ-aminobutyrate (GABA) on both conditions. Wall and Messier (2000) showed subsequently that pretreatment with an opioid kappa receptor antagonist was anxiogenic and disrupted working memory. Additional papers supporting interactions between anxiety and memory were cited by Kalueff (2007). They included demonstration of memory improvement by serotonin, whereas a decreased ability to increase serotonin is a model of anxiety.

## Serotonin and the 5-HT_2B_ receptor

Serotonin acts on many different receptors. The present paper specifically deals with the 5-HT_2B_ receptor, which is expressed in human brain (Schmuck et al., 1994; Bonhaus et al., 1995). Its mRNA expression is two times higher in freshly isolated (Lovatt et al., 2007) mouse astrocytes than in neurons (Li et al., 2012). It is necessary for consolidation of one-trial aversive learning in day-old chickens (Gibbs and Hertz, 2014) at an early stage of memory consolidation. It is also required for the therapeutic effect of serotonin-specific reuptake inhibitors (SSRIs) in major depression (Diaz et al., 2012; Li et al., 2012; Hertz et al., 2015b), a disease often accompanied by anxiety. The 5-HT_2B_ receptor in cultured astrocytes is stimulated by fluoxetine (Li et al., 2008: Qiao et al., 2015) and all other SSRIs (Zhang et al., 2010). Chronic treatment of mice with fluoxetine for 14 days up-regulates the astrocytic, but not the neuronal 5-HT_2B_ receptor, although this receptor is expressed in both cell types (Li et al., 2012; Hertz et al., 2015b). Decrease of its astrocytic gene expression parallels development of a depressive phenotype in a mouse model of Parkinson's disease (Zhang et al., 2015), and Pitychoutis et al. (2015) reported schizophrenia-like symptoms in mice lacking the 5-HT_2B_ receptor gene or treated with a receptor inhibitor. Schizophrenia is often associated with depressed mood (Fortunati et al., 2015).

## 5-HT_2B_ receptor, [Ca^2+^]_i_, glycogenolysis, glutamate, K^+^, and learning

Inhibition of learning by a 5-HT_2B∕C_ receptor antagonist (SB221284) and equipotent rescue of impaired learning by the 5-HT_2B_ receptor agonists fluoxetine and paroxetine (Gibbs and Hertz, 2014) injected intracerebrally at specific times shows the importance of this receptor for establishment of memory soon after training. The similar potency of the two drugs is important, because they have widely different affinities for SERT (Wong and Bymaster, 1995) whereas all SSRIs have similar affinity for the 5-HT_2B_ receptor (Zhang et al., 2010). Another SSRI, citalopram, counteracts spatial memory deficits (Ren et al., 2015). Mice lacking the 5-HT_2B_ receptor gene show learning disabilities (Pitychoutis et al., 2015).

**Figure 1:**
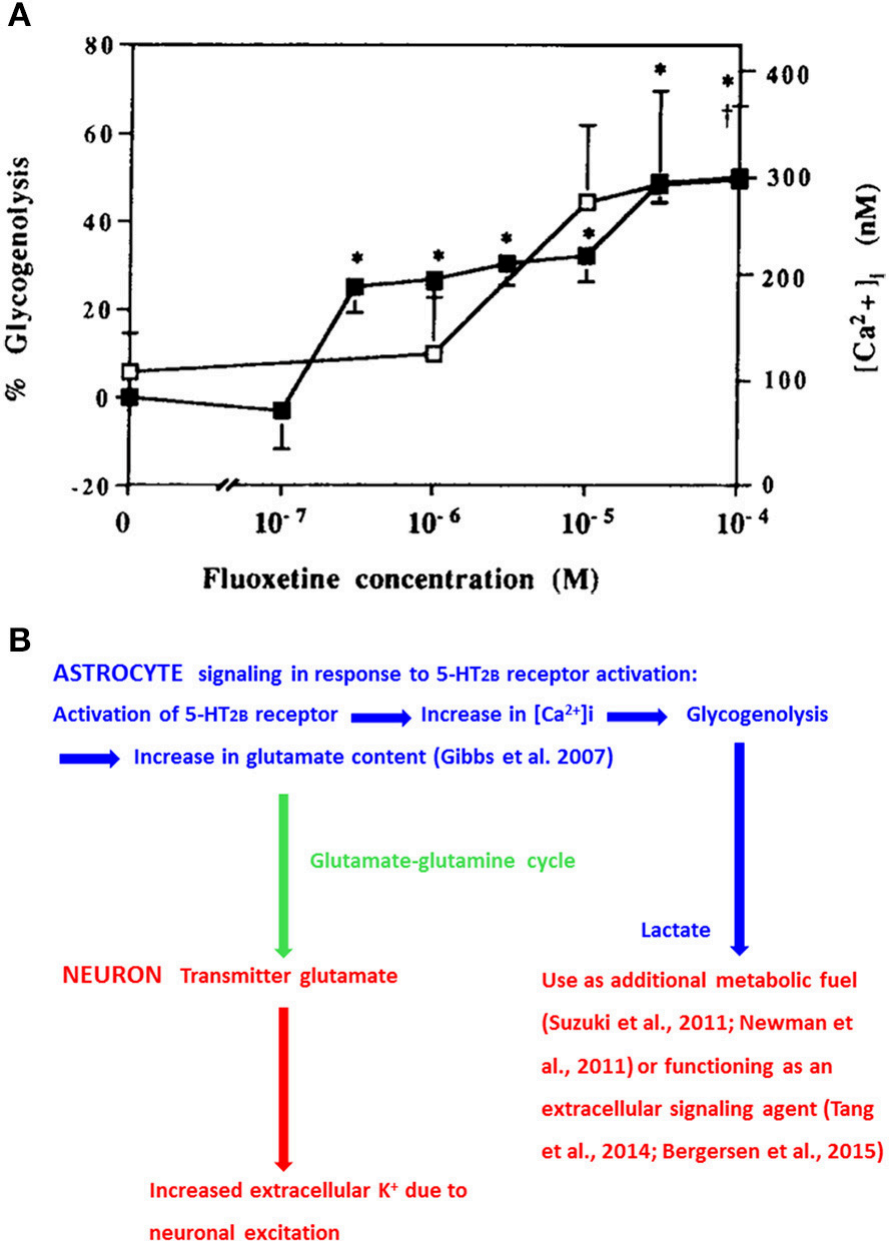
**(A)** Effects of fluoxetine concentrations between 100 nM and 100 μM on glycogenolysis (filled squares and left ordinate) and [Ca^2+^]_i_ (open squares and right ordinate) in well differentiated cultures of mouse astrocytes. Values significantly different from baseline are indicated by ^*^ for glycogenolysis and by † for [Ca^2+^]_i_ (From Chen et al., 1995). **(B)** Chart showing 5-HT_2B_ receptor-mediated effects on [Ca^2+^]_i_, glycogenolysis, and glutamate content in astrocytes (blue) and effects of glutamate transfer to neurons (green arrow) and of glycogenolysis-evoked release of lactate on neurons (red). These effects are acutely important for learning, and drug-induced chronic effects of 5-HT_2B_ receptor stimulation have therapeutic effect, also on impaired memory, in major depression (fluoxetine) and in schizophrenia (clozapine). However, as discussed in “Concluding remarks” in these situations it appears that it is a decreased effect on the receptor or on [Ca^2+^]_i_that is therapeutically effective.

Fluoxetine increases free cytosolic Ca^2+^ ([Ca^2+^]_i_) and stimulates glycogenolysis (Chen et al., 1995) with similar potency by stimulation of 5-HT_2B_ receptors (Kong et al., 2002; Figure 1A). [Ca^2+^]_i_ regulates many astrocytic functions, including gliotransmission and glycogenolysis (Gucek et al., 2012; Hertz et al., 2015a). Inhibition of glycogenolysis with DAB (1,4-dideoxy-1,4-imino-D-arabinitol) prevents 5-HT_2B_-receptor-mediated memory enhancement by serotonin or fluoxetine during the early part of memory formation after one-trial aversive learning in the day-old chicken, a precocious animal (Gibbs and Hertz, 2014). In brain both glycogen and its degrading enzyme glycogen phosphorylase are virtually confined to astrocytes (Ibrahim, 1975; Pfeiffer-Guglielmi et al., 2003). Induction of glycogenolysis by fluoxetine occurs both in our cultured astrocytes, differentiated by dibutyryl cyclic AMP and in astrocytes grown in the absence of this agent (Allaman et al., 2011). The association with increased [Ca^2+^]_i_ (Chen et al., 1995) is important because increased [Ca^2+^]_i_ is a requirement for stimulation of glycogenolysis in astrocytes (Xu et al., 2014a; Hertz et al., 2015a) as in muscle (Ozawa, 2011). In rat brain 5-HT_2_ receptor stimulation similarly induces glycogenolysis (Darvesh and Gudelsky, 2003). The enhanced glycogenolysis is accompanied by an increased lactate release (Allaman et al., 2011). This might affect neurons either by use of lactate as an additional metabolic fuel, as suggested by Suzuki et al. (2011) and Newman et al. (2011), or by lactate signaling (Tang et al., 2014; Bergersen, 2015). The signaling mechanism established by Tang et al. (2014) is, like memory (Gibbs et al., 2006; Newman et al., 2011; Suzuki et al., 2011; Gibbs and Hutchinson, 2012; Duran et al., 2013), glycogenolysis-dependent, and its signaling is specifically directed to neurons releasing noradrenaline. Noradrenaline has effects on both neurons and astrocytes (O'Donnell et al., 2012).

Glycogenolysis is also required for formation of glutamate, and its metabolite GABA (Figure 1B) in the brain *in vivo* (Gibbs et al., 2007, 2008) at a time when glutamate production must be evoked by 5-HT_2B_ stimulation (Gibbs and Hertz, 2014). It also increases uptake of glutamate into cultured astrocytes and neurons as well as release of lactate from astrocytes (Sickmann et al., 2009). Glutamate is synthesized intracerebrally from glucose. This can only occur in astrocytes, because neurons lack an enzyme, pyruvate carboxylase, needed for its synthesis (reviewed by Gibbs et al., 2008; Hertz, 2013). Glutamate is subsequently converted to glutamine and carried to neurons (green arrow in Figure 1B) in an extremely active glutamine-glutamate/GABA cycle, which also returns released transmitter glutamate to neurons after its initial astrocytic accumulation (reviewed by Hertz, 2013; Hertz and Rothman, in press). The importance of glutamate receptor activity for memory is beyond doubt (Riedel et al., 2003), and interruption of the glutamine-glutamate/GABA cycle by inhibition of either glutamine synthetase (Kant et al., 2014) or astrocytic glutamate uptake (Gibbs et al., 2004) abolishes learning. GABA is also important for learning (Kalueff and Nutt, 1996; Gibbs and Bowser, 2009), and besides its neuronal effects stimulates glycogenolysis in cultured astrocytes and brain slices (Xu et al., 2014a).

A major role of glutamate (Figure 1B) is stimulation of postsynaptic glutamate receptors, leading to increases in brain metabolism (Howarth et al., 2012) and in extracellular K^+^ concentration (Hertz et al., 2015c and references therein). Cellular re-accumulation of K^+^ includes an initial uptake mediated by the astrocytic Na^+^,K^+^-ATPase (MacAulay and Zeuthen, 2012; Hertz et al., 2015c), release of astrocytically accumulated K^+^ by Kir4.1 channels (Bay and Butt, 2012) and neuronal reuptake. The astrocytic Na^+^,K^+^-ATPase is important for learning (Moseley et al., 2007; Schaefer et al., 2011; Hertz et al., 2013; Tadi et al., 2015). Extracellular K^+^ concentrations high enough to stimulate the Na^+^, K^+^, 2 Cl^−^ co-transporter NKCC1 (>10–12 mM) also causes release of gliotransmitters (Song et al., 2014; Xu et al., 2014b; Liu et al., 2015). Both astrocytic release of glutamate (Lee et al., 2014) and ATP (Gibbs et al., 2011; Stehberg et al., 2012) are crucial for learning. Facilitation of learning by K^+^-mediated depolarization of oligodendrocytes and increased myelination at high extracellular K^+^ concentration (Roitbak, 1984) and attributed to increased ability of the myelinated axon to carry out rapid impulse conduction has recently been confirmed and characterized by Yamazaki et al. (2014).

## 5-HT_2B_ receptor, [Ca^2+^]_i_, glycogenolysis, glutamate, K^+^, and mood disorders

Fluoxetine is better known for its antidepressant effect, which in contrast to the acute stimulation of the 5-HT_2B_ receptor during learning takes several weeks to materialize. During this time many changes occur in gene expression and editing, as shown in mice chronically treated with fluoxetine. Studies in neuronal and astrocytic cell fractions freshly obtained from these mice (Lovatt et al., 2007) showed that most of these alterations occurred in astrocytes, although some neuronal changes also took place (Li et al., 2012; Peng et al., 2014; Hertz et al., 2015b). This finding suggests that astrocytes play a major role in the antidepressant effects of SSRIs (Li et al., 2012; Hertz et al., 2015b), a conclusion in agreement with results by many other authors (e.g., Ongür et al., 1998; Kugaya and Sanacora, 2005; Ongür et al., 2007; Valentine and Sanacora, 2009; Rajkowska and Stockmeier, 2013; Rajkowska et al., 2013; Bernstein et al., 2015; Hertz et al., 2015b and references therein). It is especially interesting that Bechtholt-Gompf et al. (2010) found that blockade of astrocytic glutamate uptake in rats induces signs of anhedonia (a component of depression that is easily measurable in animals) *and* impaired spatial memory.

Some of the editing changes reduced normally occurring effects of transmitters. Li et al. (2011) showed that in astrocyte cultures treated for sufficient length of time with fluoxetine, the effects on [Ca^2+^]_i_ by acute administration of several transmitters or ryanodine receptor agonists are reduced or abolished. On the other hand, the effect of an increased extracellular concentration of K^+^ was increased. Thus, chronic treatment with an SSRI diminishes or alters some of the normal responses of the 5-HT_2B_ receptor to stimulation. This might partly be explained by inhibition of capacitative Ca^2+^ entry, mediated by glycogenolysis-dependent (Müller et al., 2014) TRPC1 channels, which causes depletion of Ca^2+^ stores. Due to this inhibition refilling of depleted Ca^2+^ stores by addition of 2 mM CaCl_2_ to the medium was greatly reduced (Li et al., 2011). All effects of chronic fluoxetine administration could be replicated by TRPC1 channel antibody. However, the expression of Ca_v_1.2, a gene of an L-channel for Ca^2+^ which is stimulated by elevations in extracellular K^+^ concentrations of at least 10 mM is increased (Du et al., 2014), probably explaining the enhanced K^+^ effect on [Ca^2+^]_i_ described above. The 5-HT_2B_ receptor itself is also edited by chronic fluoxetine treatment, rapidly reducing the effects of its stimulation of the IP_3_ receptor (Peng et al., 2014). Since chronic SSRI treatment improves memory in depressed patients (Table in Krysta et al., 2015), inhibition of glutamate-induced increase in astrocytic [Ca^2+^]_i_ and thus in release of gliotransmitter glutamate (Peng et al., 2012) has no deleterious effect on learning, at least not when combined with [Ca^2+^]_i_ increase by elevation of the extracellular K^+^ concentration. In this connection it seems of considerable interest that Medina et al. (2015) described down-regulation of mRNA expression of glutamate transporters, K^+^ channels and gap junction proteins in hippocampus of patients having suffered from major depression. Most of these genes are selectively expressed in astrocytes. Abnormalities of Na^+^,K^+^-ATPase function in depressed patients have been described by De Lores Arnaiz and Ordieres (2014)

## Related astrocytic mechanisms in schizophrenia

Schizophrenia is treatable both by the dopamine antagonist haloperidol and atypical antipsychotics like clozapine, which is an antagonist at the 5-HT_2B_ receptor in the fundus of the stomach (Villazón et al., 2003). Again, acute stimulation of the 5-HT_2B_ receptor is likely to increase [Ca^2+^]_i_, glycogenolysis and glutamate formation (Figure 1B). An increase in [Ca^2+^]_i_ by stimulation of astrocytic dopamine receptors is reduced by exposure to clozapine (Reuss and Unsicker, 2001), and this seems also to be the case after clozapine activation of 5-HT_2B_ receptors. A resulting reduced production of glutamate (Figure 1B) in mice lacking 5-HT_2B_ receptors may explain a decreased content of glutamate in some brain areas (Pitychoutis et al., 2015), which may contribute to the impairment of learning.

## Concluding remarks

Activation of the astrocytic 5-HT_2B_ receptor stimulates an *increase* in [Ca^2+^]_i_, glycogenolysis, glutamate formation, and the effect of glutamate on extracellular K^+^, all of which are involved in learning (Figure 1B). However, Sibille et al. (2015) found that acute *inhibition* of Ca^2+^ signaling in astrocytes by [Ca^2+^]_i_ chelation potentiates excitatory synaptic transmission. This apparent contradiction may be explained by the complexity of astrocytic [Ca^2+^]_i_ regulation (Volterra et al., 2014). An important difference between Gibbs and Hertz (2014) and Sibille et al. (2015) is that the latter authors elicited astrocytic increase in [Ca^2+^]_i_ in response to adjacent neuronal activity during GABA_A_ receptor inhibition, whereas the former described transmitter-induced, glycogenolytic (and thus Ca^2+^-dependent) effects on learning without GABA_A_ receptor inhibition.

Drugs used for treatment of symptoms of major depression (fluoxetine) and of schizophrenia (clozapine), which included memory impairment, interfered with 5-HT_2B_ receptor-activated functions, but in different manners: the SSRI fluoxetine edited and thereby reduced some normal effects of this receptor, whereas clozapine caused a decrease in [Ca^2+^]_i_. This effect is consistent with the enhancement of excitatory synaptic transmission described by Sibille et al. (2015). Disposition to both major depression and schizophrenia is probably inborn, and perhaps these patients display quantitative and/or qualitative abnormalities in 5-HT_2B_-mediated signaling, which might also affect learning processes. In agreement with this notion 5-HT_2B_ receptors play a major role during brain development (Lauder et al., 2000).

## Author contributions

All authors planned or carried out reviewed experiments. YC and LH wrote the paper and MEG edited it.

### Conflict of interest statement

The authors declare that the research was conducted in the absence of any commercial or financial relationships that could be construed as a potential conflict of interest.
